# Fano Resonance-Associated Plasmonic Circular Dichroism in a Multiple-Dipole Interaction Born–Kuhn Model

**DOI:** 10.3390/s24237517

**Published:** 2024-11-25

**Authors:** Wanlu Bian, Guodong Zhu, Fengcai Ma, Tongtong Zhu, Yurui Fang

**Affiliations:** 1 School of Physics, Dalian University of Technology, Dalian 116024, Chinayrfang@dlut.edu.cn (Y.F.); 2 School of Physics and Electronic Information Engineering, Chifeng University, Chifeng 024000, China; 3 Department of Physics, Liaoning University, Shenyang 110036, China

**Keywords:** circular dichroism, Fano resonance, intrinsic chirality

## Abstract

Plasmon chirality has garnered significant interest in sensing application due to its strong electromagnetic field localization and highly tunable optical properties. Understanding the effects of mode coupling in chiral structures on chiral optical activity is particularly important for advancing this field. In this work, we numerically investigate the circular dichroism (CD) of elliptical nanodisk dimers arranged in an up-and-down configuration with a specific rotation angle. By adjusting the inter-particle distance and geometric parameters, we introduce the coupling between dipole and electric hexapole modes, forming an extended Born–Kuhn model that achieves strong CD. Our findings show that the coupling of dipole modes with electric hexapole modes in elliptical nanodisks can also show obvious Fano resonance and a strong CD effect, and the structure with the largest Fano asymmetry factor shows the highest CD. In addition, CD spectroscopy is highly sensitive to changes in the refractive index of the surrounding medium, especially in the visible and near-infrared regions, highlighting its potential for application in high-sensitivity refractive index sensors.

## 1. Introduction

Chirality, the property by which an object does not match its mirror image, has attracted significant research interest due to its prevalence in nature and biology [1]. Measuring chirality is valuable in fields such as medical diagnostics [2], analytical chemistry [3,4], and molecular biology [5,6]. However, natural molecules typically exhibit weak optical activity (OA), making their detection challenging. In contrast, plasmonic chirality offers strong OA and highly localized electromagnetic fields, showing great potential for sensing chiral molecules. Plasmon resonance, sensitive to the geometry and material composition of structures, allows for broad OA tunability across ultraviolet to near-infrared wavelengths. As a result, various chiral plasmonic structures, such as helical coils [7,8,9,10], chiral crescents [11,12,13,14], double-twisted rings/rods [15,16,17,18,19,20], chiral particles [21,22,23,24] and DNA-based assemblies [25,26,27,28] have been experimentally prepared, all exhibiting strong OA.

Most of the studies mentioned above utilize the plasmonic Born–Kuhn model to elucidate the optical activity of plasmonic chiral structures, forming the foundation for the design of nanostructures with enhanced optical activity. However, this model is primarily confined to interactions involving dipole resonances. In recent years, there has been a growing interest in exploring the higher-order resonances of plasmonic structures [29]. The higher-order chirality of the electric quadrupole mode has been experimentally measured and quantitatively analyzed [13,30]. Additionally, numerous chiral structures with subwavelength dimensions have been fabricated [31,32,33], demonstrating significant potential for various applications. However, the underlying mechanism of chirality extends beyond what can be explained by the Born–Kuhn model [34], which is limited to dipole resonance. In particular, the Fano resonance caused by the coupling of electric dipole and electric hexapole modes has been explored theoretically, but the optical activity caused by this coupling has not been explored [35]. Thus, an extended plasmonic Born–Kuhn model is essential for understanding higher-order plasmonic chirality and enhancing chiral optical responses.

In this work, we investigate an extended Born–Kuhn model consisting of elliptical nanodisk heterodimers with different geometrical sizes, allowing for the coupling of dipolar and multipolar modes at the resonant wavelength. The interaction between the dipole resonance of the small nanodisk and the higher-order modes of the large nanodisk induces Fano resonances that lead to a strong CD response. Furthermore, the CD response generated by the high-order resonant coupling is as strong as that of the dipolar Born–Kuhn model, which is beyond the theoretical framework of mixed electric and magnetic dipole interactions. With a sweep of the geometric parameters, we also found that the CD response of the structure is closely related to the Fano resonance, which can flexibly regulate the chiral optical response of structures. At the same time, the structure also exhibits an excellent sensing performance, which may have promising applications in sensing [36].

## 2. Results and Discussion

**Figure 1:**
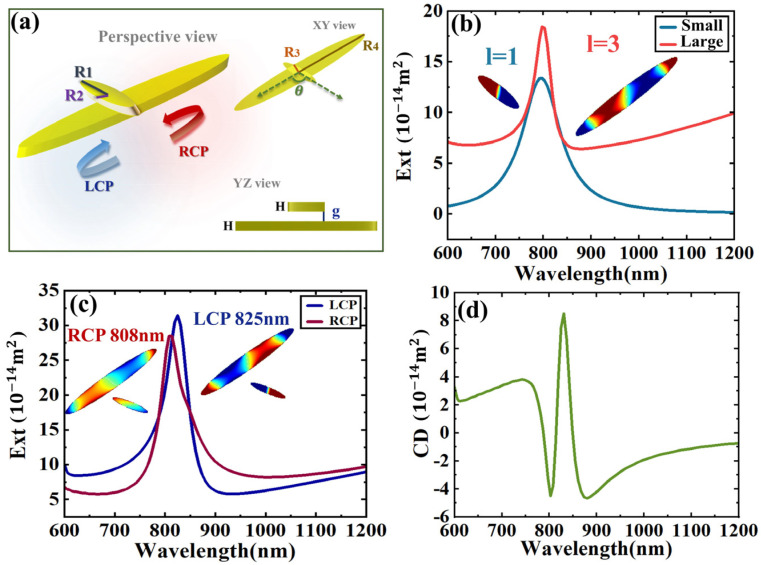
Born–Kuhn model composed of two nanodisks. (**a**) Structure scheme, R_1_ = 100 nm, R_2_ = 22.5 nm, R_3_ = 50 nm, R_4_ = 400 nm, the thickness of the two particles H is 50 nm, the gap g between the upper and lower is 50 nm, the angle is 120°, and the light incident is perpendicular to the cross plane of the two nanodisks. (**b**) The extinction cross-section of individual nanodisks. The insets show the charge distributions of the nanodisks at the resonance wavelength of 800 nm. (**c**) The extinction cross-section of the structure excited by LCP and RCP. The insets show the surface charge distributions at the corresponding peaks. (**d**) CD spectrum.

We employed two gold elliptical nanodisks with distinct dimensions to construct a Born–Kuhn model, as illustrated in Figure 1a. The smaller particles have a semi-major axis (R_1_) of 100 nm and a semi-minor axis (R_2_) of 22.5 nm. The larger particle has a semi-major axis (R_4_) of 400 nm and a semi-minor axis (R_3_) of 50 nm. Both particles possess a thickness (H) of 50 nm, are separated by a distance (g) of 50 nm, and form an angle of 120°. The model is placed in a cubic cell with a side length of 2000 nm and a refractive index of n = 1. The permittivity of gold nanodisks are adopted according to Johnson and Christy’s works [37]. We used the commercial software Ansys Lumerical FDTD (Version 2020 R2.4) to perform our calculations. The light source is set as two beams of orthogonal linearly polarized light with a phase difference (±90°), corresponding to the left-handed and right-handed polarization states, respectively, and the incident wavelength range covers 600 nm to 1200 nm. The light source irradiates the chiral structure vertically along the Z axis to stimulate strong electromagnetic interactions. In order to ensure the accuracy of the simulation, the perfectly matched layer (PML) of a stretched coordinate type is used to absorb boundary reflection to ensure the accuracy of the simulation. For the acquisition of the extinction cross-section, two sets of analysis data results are added together. The two sets of data come from monitors placed inside and outside the light source, respectively. The former is responsible for monitoring the absorption effect, and the latter records the scattering effect.

Figure 1b shows the extinction cross-section of the individual nanodisks, where the blue curve corresponds to the smaller one and the red curve represents the larger one. The inset depicts the surface charge distribution of the nanodisks at the resonance wavelength 800 nm. It is observed that under the excitation of an incident light with a wavelength of 800 nm, only the dipole resonance is excited in the small nanodisk. In contrast, the large ones, due to their high aspect ratio, exhibit an *l = 3* electric hexapole resonance. The resonance wavelengths of both structures overlap. Figure 1c shows the Born–Kuhn structure response under left-handed circular polarized light (LCP) and right-handed circular polarized light (RCP), respectively, with normal illumination. The blue curve corresponds to LCP excitation, with the resonance wavelength of the nanodisks dimer at 825 nm, while the red curve corresponds to RCP excitation, with a resonance wavelength of 808 nm. This indicates that the interaction between the electric dipole *l* = 1 and electric hexapole resonance *l* = 3 causes the extinction cross-section of the structure to exhibit a distinct Fano line shape. Figure 1d is the CD spectrum, derived from CD = Ext_LCP_ − Ext_RCP_. It can be seen that the extended Born–Kuhn model, resulting from the coupling between electric dipole and electric hexapole resonances, exhibits a pronounced chiral response. Moreover, the CD spectrum peak shows a very narrow linewidth, highlighting its potential for applications in sensing and single-wavelength circular polarization wave plates.

**Figure 2:**
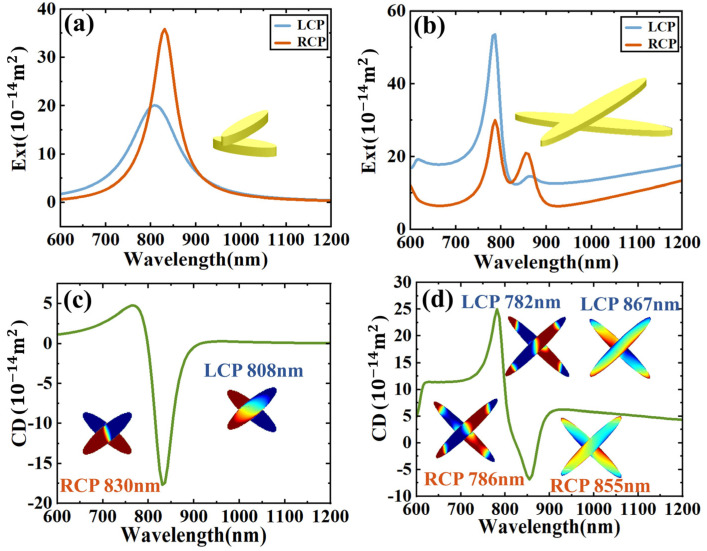
(**a**) Extinction cross-section of a dimer composed of small dimers. (**b**) The nano-configuration of a dimer composed of two large particles. CPL excites the extinction cross-section of the two large particles. (**c**) The extinction CD spectrum and the charge distribution of the upper and lower particles after excitation of the corresponding formant. (**d**) The extinction CD spectrum and the charge distribution of the upper and lower particles after the LCP and RCP formant excitation.

In order to investigate the CD response properties of the structure, the dimers consisting of same-sized nanodisks are also explored. The size parameters for nanodisks are the same as in Figure 1a. Figure 2a,b show the extinction cross-section of the smaller and larger dimers, respectively. From the spectra, we can see that with the same gap, the smaller dimer exhibits a single resonance, while the larger dimer shows two distinct resonance peaks. This indicates that the larger dimer experiences stronger interactions between the particles, leading to the splitting of the resonance into two peaks. The surface charge density distribution reveals that the smaller dimer excites a hybrid mode under circularly polarized light, with LCP exciting the antibonding mode and RCP exciting the bonding mode. In contrast, for the larger dimer, the coupling of its electric hexapole mode is more complex, making it difficult to distinguish clearly between bonding and antibonding modes. Figure 2c,d show the CD spectra of the two dimers. Both of them show very obvious chiral optical responses. For the smaller dimer, the CD value is mainly negative and for the larger dimer the CD value is mainly positive. Meanwhile, the smaller dimer shows a stronger peak at the longer wavelength, but the larger dimer shows a stronger peak at the shorter wavelength. From the charge distributions, we have already seen that the hybrid resonances of dipole–multipole strength are larger at the corresponding peaks (830 nm for smaller dimer and 782 nm for the larger dimer). So, we expect that a dimer composed of one smaller disk and one larger disk would have stronger interactions between the two particles and strong resonance at both peaks.

**Figure 3:**
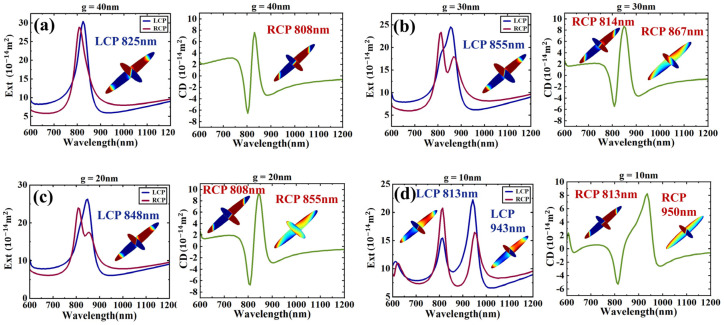
(**a**) The extinction cross-section and the corresponding CD spectrum for the distance *g* between the two particles at 40nm, and the illustration shows the charge distribution at the corresponding resonance. (**b**) *g* = 30 nm. (**c**) *g* = 20 nm. (**d**) *g* = 10 nm.

To further investigate the impact of the interaction between the two nanodisks in the dimer on optical activity, we reduced the gap between the nanodisks to achieve stronger coupling. Figure 3a shows that when the gap is reduced to 40 nm, LCP excites the electric dipole–hexapole bonding mode at 825 nm, while RCP excites the electric dipole–hexapole antibonding mode at 808 nm. This configuration results in a stronger CD response compared to the *g* = 50 nm case shown in Figure 1. In Figure 3b, when the gap is further reduced to 30 nm, the dimer exhibits a dipole–hexapole bonding mode at 855 nm under LCP excitation, while the extinction curve for RCP excitation splits into two peaks. Although the interaction in the gap is strong enough, the peaks only show obvious splitting when the upper and lower nanodisks undergo phase jumps under RCP excitation. In Figure 3c, when *g* is reduced to 20 nm, it shows an extinction cross-section and CD spectrum similar to when *g* = 30 nm. In Figure 3d, when *g* is reduced to 10 nm, the spectra for both LCP and RCP excitation split into two peaks, indicating strong dipole–hexapole interaction modes. For LCP, the antibonding mode appears at 813 nm, while the dipole–hexapole bonding mode is at 943 nm. For RCP, the antibonding mode is at 813 nm, and the bonding mode is at 950 nm. This splitting complicates the analysis of the Fano resonances at these peaks. However, the asymmetry between the peaks becomes increasingly distinct and pronounced.

**Figure 4:**
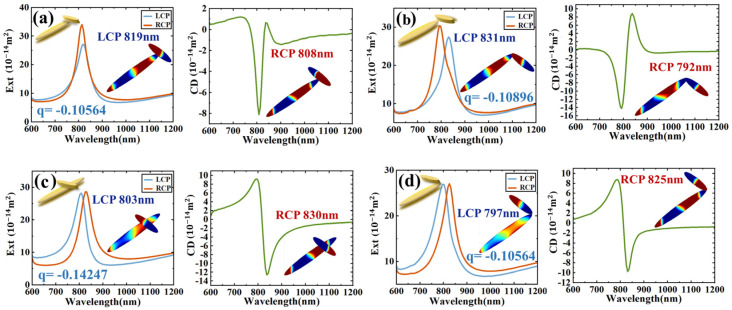
(**a**) The central dot of the small nanodisk overlaps with the vertex of the large nanodisk. (**b**) The vertex of the left end of the small nanodisk overlaps with the vertex of the large nanodisk. (**c**) The center point of the small nanodisk overlaps with the middle of the center point and the vertex of the large nanodisk. (**d**) The vertex of the right end of the small nanodisk overlaps with the vertex of the large nanodisk.

By keeping the larger nanodisk in place and moving the smaller nanodisk, the overall configuration of the dimer changes, as shown in the inset of Figure 4. The smaller nanodisk moves along the long axis of the larger one. When the center point of the smaller one coincides with the vertex of the larger one, the extinction cross-section and CD spectrum of the dimer are as shown in Figure 4a. At LCP-819 nm, the dimer presents a dipole–hexapole bonding mode, and at RCP-808 nm, the dimer presents a dipole–hexapole antibonding mode. When the small particle is moved along the long axis of the large particle so that the center point of the small particle overlaps with the middle of the center point and the vertex of the large particle, the dimer extinction cross-section and CD spectrum are as shown in Figure 4b. At LCP-803 nm, the dimer presents a dipole–hexapole bonding mode, and at RCP-830 nm, the dimer presents a dipole–hexapole antibonding mode. When the vertex of the left end of the small particle overlaps with the vertex of the large particle, the dimer extinction cross-section and CD spectrum are as shown in Figure 4c. At LCP-831 nm, the dimer presents a dipole–hexapole bonding mode, and at RCP-792 nm, the dimer presents a dipole–hexapole antibonding mode. When the vertex of the right end of the small particle overlaps with the vertex of the large particle, the dimer extinction cross-section and CD spectrum are shown in Figure 4d. At LCP-797 nm, the dimer presents a dipole–hexapole antibonding mode, and at RCP-825 nm, the dimer presents a dipole–hexapole antibonding mode. The Fano line shape in Figure 4c,d shows an obvious opposite trend.

To quantitatively show the Fano-assisted CD, we chose the LCP scattering spectrum peak to perform the Fano fitting with σ=qωres2+E−Eres2ωres22+E−Eres2 (where q is the Fano asymmetry factor, and $\omega_{res}$ is the peak width of resonance energy). The Fano asymmetry factors are obtained as q=−0.10564,−0.14247,−0.10896,−0.12296 for Figure 4a–d, respectively. It is evident that the structure exhibiting a greater absolute value of the Fano asymmetry factor in its spectrum tends to have a higher CD value [38].

**Figure 5:**
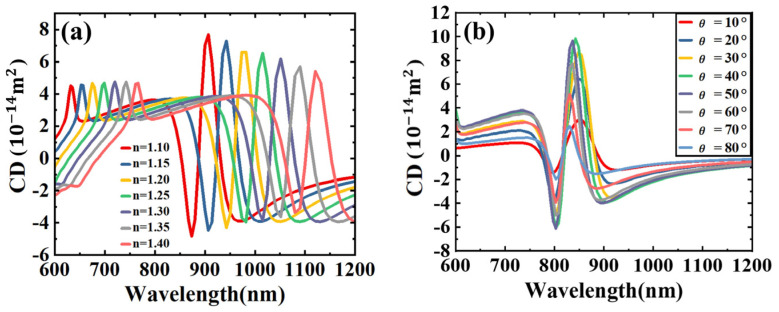
(**a**) The CD spectrum after the surrounding medium environment n increased from 1.1 to 1.4. (**b**) The CD spectrum when the angle between the two particles increased from 10° to 80°.

To investigate the effect of the surrounding dielectric environment on the plasmonic characteristics of the Fano resonance, we calculated the chiral optical response of the structure under different surrounding media. Figure 5a shows that as the refractive index of the surrounding medium increases, the CD spectrum undergoes a significant red shift. This observation indicates that the structure is highly sensitive to changes in the dielectric environment, making it suitable for use as a highly sensitive sensor. In Figure 5b, we also examined the asymmetry of the dimer by varying the angle between the two nanostructures. The results show that the changes in the angle led to significant variations in the CD value of the dimer. These findings demonstrate the sensitivity of the dimer’s optical response to both the surrounding dielectric environment and its geometric configuration, highlighting its potential for applications in sensing and the regulation of polarized light.

## 3. Conclusions

In summary, we systematically investigated a tunable Fano resonance for plasmonic nanostructures with an extended Born–Kuhn model, which consists of two elliptical nanodisks of varying sizes. Due to the coupling of the hexapolar resonance of the larger nanodisk with the dipolar resonance of the small nanodisk, a tunable Fano resonance within a wide range of spectra from visible light to mid-infrared spectrum is achieved. The spectral peak and the CD response of the Fano resonances can be modulated within a wide range by changing the geometry parameters of the dimer. By providing a comprehensive investigation of the CD properties of nanodisks and their dependence on various parameters, this work contributes to the understanding of nanoscale optical phenomena and provides valuable insights for the development of advanced CD-based sensing technologies.

## Data Availability

The data that support the findings of this study are available from the corresponding author upon reasonable request.
